# Popular Medicine and Empirics in Greece, 1900–1950: An Oral History Approach

**DOI:** 10.1017/mdh.2016.57

**Published:** 2016-10

**Authors:** Violetta Hionidou

**Affiliations:** Newcastle University, School of History, Classics and Archaeology, Armstrong Building, Newcastle upon Tyne NE1 7RU, UK

**Keywords:** Greece, medicine, empirics, popular healing, oral history

## Abstract

Western literature has focused on medical plurality but also on the pervasive existence of quacks who managed to survive from at least the eighteenth to the twentieth century. Focal points of their practices have been their efforts at enrichment and their extensive advertising. In Greece, empirical, untrained healers in the first half of the twentieth century do not fit in with this picture. They did not ask for payment, although they did accept ‘gifts’; they did not advertise their practice; and they had fixed places of residence. Licensed physicians did not undertake a concerted attack against them, as happened in the West against the quacks, and neither did the state. In this paper, it is argued that both the protection offered by their localities to resident popular healers and the healers’ lack of demand for monetary payment were jointly responsible for the lack of prosecutions of popular healers. Moreover, the linking of popular medicine with ancient traditions, as put forward by influential folklore studies, also reduced the likelihood of an aggressive discourse against the popular healers. Although the Greek situation in the early twentieth century contrasts with the historiography on quacks, it is much more in line with that on wise women and cunning-folk. It is thus the identification of these groups of healers in Greece and elsewhere, mostly through the use of oral histories but also through folklore studies, that reveals a different story from that of the aggressive discourse of medical men against quacks.

‘The lies of the quacks collapse. There were neither miracles, nor healings. It is time for the authorities to intervene.’^1^ Thus read a headline in a Greek national newspaper in 1930. The article below it referred to an untrained healer, Monk Gymnasios, who had attracted the attention not only of thousands of people who hoped to be cured but also, repeatedly, of the authorities. He dominated the news in 1930–31, when, time after time, he was arrested, imprisoned, released and removed to a different part of the country, where, invariably, he was awaited by thousands of people who wanted to consult him and be healed by him.^2^ While he owed his reputation, at least initially, exclusively to word of mouth, newspaper reports such as that cited above inevitably contributed to it. Nevertheless, Gymnasios, whose therapies were a mix of herbal recipes and religious incantations, emphasised that ‘I do not pretend to be a physician. I do not steal from the people. I do not take money.’^3^ He never remained imprisoned for long, partly because he received no remuneration from his patients and partly because of his enormous popularity. However, in 1932, he stopped practising because of the continual persecution he suffered and because he failed in his efforts to obtain a licence;^4^ like all irregulars, he lacked training and did not possess the necessary licence to practise.

Although the distinction between those who could and could not practise medicine legally in Greece was codified as early as 1834, unlicensed practitioners continued to practise well into the twentieth century; while Gymnasios was the most famous unlicensed practitioner of inter-war Greece, he was only one of many practising at the time. But was he a typical representative or, rather, was he exceptional? Who were these unlicensed practitioners and why did they practise illegally, if not for profit? How did their patients know about them and why did they choose them – if, indeed, they did – instead of physicians? What were the mechanisms that enabled their survival beyond the first half of the twentieth century? In contrast with untrained practitioners in the West, we know very little about those in Greece beyond their certain existence. This paper will therefore explore the nature of irregular practice in Greece in the first half of the twentieth century, offering an alternative to what we have come to expect from Western accounts of quackery; but one that, nevertheless, shares many common attributes with Western accounts of cunning-folk, wise women and popular healers.

A significant range of history of medicine studies has focused on the plurality of medical and healing practices from the eighteenth century to the present.^5^ Such studies reveal the co-existence of the healing practised by learned physicians with alternative medical practices such as homeopathy and hydropathy, among others. But, as Robert Jütte points out, the professionalisation of medicine during the nineteenth century critically influenced the hardening of conceptual boundaries between medicine and its alternatives.^6^ However, Roger Cooter has argued that, even at the turn of the twentieth century, the division between quacks and learned medics was not always clear,^7^ while, more recently, Takahiro Ueyama has noted that, beyond the official (and dominant) narrative of the medical profession, medical practice, in fact, experienced significant commercialisation in late nineteenth-century London, so blurring rather than better defining the boundary between the quack and the physician.^8^ And, just as in Britain, in the USA the professionalisation of medicine neither eliminated quackery nor made it easier to identify;^9^ indeed, the consensus among scholars is that, in the USA, quackery, or plurality, was never eliminated and, while better controlled in the twentieth century, is ‘still as popular as ever’.^10^

Studies in this area thus demonstrate the existence of non-learned medical practitioners alongside their learned counterparts, at least from the eighteenth century to the present day. Such practitioners, however, differed enormously in terms of their methods and their relationships with the learned physicians, the state and the patients from place to place and over time. Nevertheless, there are some common elements to be found in virtually all Western histories of non-learned medical practitioners: first, that profit was the main driver for their practice; and, second, that from the eighteenth to the twentieth century the major means of communicating with their ‘patients’ was advertising, in which they constantly adapted their therapeutic promises in response to legislative and other reforms.^11^ Already, in the 1970s, Matthew Ramsey had distinguished between the quacks – whose practices included the elements outlined above – and the popular healers, who were ‘part of the local village culture’.^12^ Yet, only occasionally have the grassroots health care practices and the reactions and adaptations to change of the latter been studied. Francesca Moore, for instance, has investigated such issues in a working-class Lancashire community at the turn of the twentieth century,^13^ presenting evidence of the ‘survival of a localised pattern of alternative care’ that was embedded in the local working-class culture and which, she argues, was partly responsible for the slow acceptance of orthodox medicine by that community.^14^ Prior to Moore, Owen Davies pointed to the lack of attention shown by historians to the cunning-folk and the role they played as medical providers in nineteenth-century England, identifying as reasons for their enduring popularity not only the fact that orthodox medicine ‘continued to be ineffective into the twentieth century’ but also that witchcraft continued to be seen ‘as a source of illnesses’.^15^

The perspective offered in the works of Davies and Moore is different from that of much other work on alternative medicine partly because of the sources these historians employed: namely, oral history and ethnographic accounts. These are also the main sources utilised in this paper. Oral histories constitute a significant and valuable source that throws light on how unlicensed practitioners – and physicians – were viewed and employed by ordinary people – primarily in terms of determining why, how and when they consulted unlicensed practitioners – and can assist in elucidating physicians’ views on unlicensed practitioners. Such information cannot be obtained from the writings of physicians in medical journals and thus these histories offer a significant corrective to the official and the physicians’ point of view. The interviews utilised in this paper were conducted by the author over a number of years: twenty-eight interviews on Mykonos in 1994, twenty-two on Chios in 2000, twenty-two on Syros in 2001 and nine in 2011 in Athens and on Chios.^16^ The interviewees ranged in age from 71 to 101 and therefore their recorded experiences span as far back as the early decades of the twentieth century. The research aims of which the interviews formed a part varied over time and, therefore, the information provided by interviewees on healers also varied in scope and detail. Significantly, however, the last set of interviews focused primarily on physicians and their experiences. In none of the interviews was an interviewee asked directly about unlicensed practitioners or physicians; rather, such information was volunteered by interviewees without prompting.

A further source significant for examining the presence and practice of unlicensed practitioners is folklore accounts. Because popular medicine was seen not only as a significant element of Greek rural life and tradition but also as a remnant of ancient Greek medical practices, information on it was intensively collected, especially in the twentieth century. While the accounts of the first half of the century come primarily from professional folklorists, later accounts were collected by a variety of individuals,^17^ and the majority of the Greek publications cited here make use of folklore material and oral accounts collected by the publications’ authors. A significant example of such work is the folklore project undertaken by undergraduate students of Athens University, who, since 1964, have collected accounts from their own places of origin and submitted their manuscripts as part of their course assessment.^18^ Such accounts are of particular significance because the illiteracy of the majority of unlicensed practitioners and the illegality of their practice meant that very little written evidence has been left behind by them or by the state.^19^ In his discussion of this source material, David Gentilcore has questioned both the reluctance of historians of medicine to utilise such ethnographic accounts and, contrastingly, the ways in which such material has been used.^20^ In the former case, the majority of the reasons offered by historians to justify their reluctance to engage with these sources do not, in fact, apply to early twentieth-century Greece. For example, an objection often raised is the ease or otherwise of distinguishing between popular and professional medicine;^21^ but in Greece their differences were very apparent by the twentieth century, as they were also in late nineteenth-century Italy. Ramsey has pointed to the bias that can affect such collections when the investigator has looked specifically for ‘folkloric’ activities,^22^ a problem overcome here by the juxtaposition of folkloric evidence against other sources, such as oral histories and anthropological works. In what follows questions regarding the identities of the unlicensed practitioners, their reasons for undertaking such work and the factors that operated in their survival beyond the mid-twentieth century in Greece are addressed in advance of a discussion of how these findings compare with what we know from similar studies of unlicensed practitioners and healers in the West.

## 1 Naming the Untrained Healers

As elsewhere, a variety of names was used in Greece to describe untrained practitioners.^23^ These terms, which might depend on the type of services provided, included: *empeirikos* or *praktikos* [empirical, someone who learns his/her art empirically]; *giatrina* or *giatrissa* [female ‘doctor’]; *magos* [magician]; *agyrtes*; and *kompogiannites*.^24^ The last two terms refer to wandering, rather than sedentary, healers and correspond to Ramsey’s itinerants.^25^ Comparatively little is known about them and their practice, which seems to have been curtailed by the end of the nineteenth century and, consequently, they will not be examined here.^26^ While the meanings of some of these terms have changed over time, they have also varied according to their user: so, for example, while the term *empeirikos* had a positive connotation for most of the rural lay population, among physicians it was used pejoratively.^27^ Because of the impossibility of determining exactly what each of the descriptive terms found in the existing sources encompasses in terms of practice, all sedentary healers, upon which this paper will focus, will be referred to here, interchangeably, as either empirics or ‘popular healers’ (although this term is not one used in Greek, these practitioners’ work was referred to in Greece as ‘Popular Medicine’ [*Laike Iatrike*]).^28^ These two names are used only for reasons of simplicity and their use should not be taken to represent the existence of a uniform practice among popular healers in Greece, since the opposite was very much the case.^29^

Despite the ambiguity in terminology noted above, untrained sedentary (that is, resident) practitioners tended to specialise in specific illnesses or conditions and, as a result, it was usual for a given geographical area to boast a number of practitioners each dealing with different ailments.^30^ These might include: bone-setters;^31^ *giatrines*, who would deal primarily with children’s and women’s ailments;^32^ healers dealing specifically with jaundice or with erysipelas; and others specialising in the removal of the ‘evil eye’.^33^ Most sedentary healers apparently combined religious/magical elements with practical healing – as discussed below – using each to a different degree.^34^ Even on occasions where no religious or magical elements were directly employed, however, the presence of religion was emphasised.^35^ In terms of practical measures, empirical practitioners relied primarily on herbal remedies,^36^ and there is no evidence of alternative medical practices such as homeopathy, mesmerism and Thomsonian medicine acquiring any significance in Greece until the 1950s; therefore, this paper does not engage with such practices. Hydropathy, in contrast, existed from the creation of the Greek state, but as it was, from the start, associated with the state and trained physicians it is not considered in this paper.

Sedentary healers were both women and men, and included priests or monks:^37^ since healing was embedded in religious practice, there was no contradiction between the two. Aspects of spiritual healing included charms, incantations [*xorkia*], blessings [*euhes*], magical spells and prayers,^38^ while many empirics reputedly owned an iatrosophic text [*iatrosophia*/*giatrosophia*], which raised their prestige.^39^ These hand-written healing manuscripts were written in vernacular Greek and listed diseases and their therapies, including incantations.^40^ Christos Papadopoulos has emphasised that such texts expressed ‘a vernacular philosophy of healing’ that did not differentiate between the pagan and the Christian; or, rather, I would argue, they brought together the pagan, the Christian and the magical beliefs of the society they represented.^41^ Both Papadopoulos and Patricia A. Clark, as well as a majority of Greek authors dealing with such healing manuscripts, stress the links between these manuscripts and Ancient Greek medicine.^42^ Moreover, it is apposite to note that Greek populations were under Ottoman rule for centuries, in consequence of which it would seem highly likely that the popular healing tradition of the Greeks – as it survived to the twentieth century – and that of Islam were strong influences upon each other.

## 2 Acquiring the Art of the Empiric

Almost invariably, the skills required of an empiric were transferred through generations within families, and thus were ‘inherited’ or learned from the parent or sometimes the grandparent.^43^ Therefore, the option of becoming an empiric was not, generally, open to all.^44^ In some exceptional cases, however, a few individuals learned the vocation from a non-related empiric to whom they were apprenticed.^45^ The strong emphasis in the oral and written accounts on the ‘inherited’ nature of the art suggests that empirics who mentored non-relatives did so either because they were childless or because they had no children wishing to learn.^46^ In addition to the instruction that empirics received, it is evident that most also tried to expand their knowledge throughout their lives, mostly through observation and experimentation.^47^

The learning required of an apprentice empiric was crucially dependent upon the existence or creation of a strong relationship between apprentice and empiric. Such relationships were created only in the empiric’s older years;^48^ for younger practitioners, secrecy was a characteristic both of the ‘occupation’ in general and of the ownership of an iatrosophic book. Although those scholars who have discussed empirics have mentioned the issue of secrecy, none has offered a possible explanation except in cases that involved incantations and spells;^49^ in such cases, passing on the knowledge was considered to render the magic ineffectual for the one who passed it on and effectual only for the receiver.^50^ A similar reason was offered by one of my interviewees, who possessed the knowledge of an incantation that removed the ‘evil eye’ cast upon someone. Only the interviewee, among all her siblings, received knowledge of the charm from her mother and, choosing among all her children, she transferred that knowledge to her son. As she explained, the charm would cease to be effective if either she or her son were to divulge its secret.^51^ As all manuscript medical books included such charms and incantations, this rule of secrecy applied to them too. Such an explanation accounts well for the observed secrecy of the healing arts and for the practice of passing them on only to the most favoured and possibly most ‘talented’ or spiritual child, but fails to explain the employment of the same level of secrecy for all kinds of healing. It might be that all healing practices, whether ‘magic’, religious or empirical, were viewed as truly enmeshed and not differentiated as such by either the healers or their patients. On the other hand, it is perhaps plausible that restrictions were placed upon the dissemination of healing knowledge because this was a profitable profession that ran within families. It made sense, in order to retain the benefits of such profits (see below) within the family, to exercise discretion by passing on the art to one select individual. Furthermore, such strict control of the dissemination of such knowledge, and the consequent control of the numbers in the ‘profession’, ensured that the market would not become saturated with empirics.

## 3 Reasons for Becoming an Empiric

In Greece, at this time, it was illegal to practise healing without a licence. This raises the question of why someone would become an empiric, given that the consequences were persecution and possible imprisonment. In fact, empirics very rarely practised healing as their prime occupation, at least from the mid-nineteenth century onwards.^52^ This applied both to men, who would have a main occupation, and to women, who would have only rarely had a formal occupation in rural pre-Second World War Greece.^53^

The prime reason for the practice of ‘quackery’ in the West was income generation, and it seems an obvious reason in this case as well.^54^ However, most scholars who discuss Greek healers point out that they did not demand (monetary) payment, although it is noted that people would offer payment in kind: ‘[T]heir payment was always various produce…eggs, chickens and the rest.’^55^ When healing is referred to as a profitable pursuit, the clarification is made that the empiric would ‘receive wheat, barley, corn, almonds, clothes etc.’ for his/her services.^56^ Such rare references to monetary payments as exist come from the early nineteenth century: for example, a cash exchange is mentioned in an 1825 extract from the cross-examination of an empiric accused of his patient’s death,^57^ and further references from the islands of Sifnos and Melos in 1838 and 1840, respectively, show empirics documenting the monetary settlements they expected to receive.^58^ The physician G. Fasoulakes, who held, unsurprisingly, somewhat negative views regarding empirics, emphasised the income-generating part of their work, especially the selling of the ‘medicinal preparations’ that they prescribed.^59^ Fiction writers also emphasised the empirics’ focus on enrichment. A popular novella published in 1903 and written from the perspective of a *giatrissa* emphasises the paramount significance to the practitioner – the Murderess – of the ‘payment’ she received for her services, although the payment was only partially monetary.^60^ Similarly, the significance of the payment was emphasised by an interviewee whose mother was a bone-setter. The interviewee mentioned in several separate instances the ‘payment’ that his mother received: ‘my mother [was a] widow, [a] very old woman[;] she knew some pseudo medical [practices] and they would give her something [payment]…. They would give her some broad beans, some oil.’^61^ Yet another individual, writing in his memoirs, noted that his grandfather offered ‘help for free’, describing an incident in which his grandfather was asked about his fee by the patient’s husband and replied that he was not practising for the money; twenty days later, however, the fully recovered patient sent the empiric a quantity of local produce in appreciation of his help.^62^ In inter-war Thrace a *giatrissa* claimed that she practised healing for ‘her soul’ and was thus not paid in money, but was allowed to take a symbolic minimal payment that she would use to light a candle in the church.^63^ Marlene Sue Arnold’s interviewee, an empiric, emphasised in the mid-1970s that he neither took payment nor accepted gifts, while pointing out the materialism of contemporary physicians.^64^ Still more recently, an interviewee who sought the help of an empiric in the late 1990s explained that the empiric asked for ‘whatever she had the pleasure to give, he did not say he did not want anything, he said to me whatever you want’, and the interviewee gave a small symbolic monetary payment.^65^ However, the practitioner told her that some of his clients made significant gifts: one expatriate offered the practitioner his car.

It is clear that practitioners did enjoy some kind of revenue from their practice, although, almost certainly, such revenue was monetary only occasionally. It was, rather, primarily in kind and flexible in nature, given only when healing occurred, and corresponded to the economic and social standing of the healed. As the majority of healing took place in small communities which enjoyed little anonymity, expectations of appropriate gifts were very well informed and in all probability closely fulfilled.^66^ Moreover, ‘gifts’ of healing would create an ‘obligation’ which invariably would be returned in due course, as local rural economies were based on systems of balanced reciprocity.^67^ Although it could be argued that such gifts occurred in such economies because these were not monetised, this is unlikely to be the only reason. The great diversity of local economies in the first half of twentieth-century Greece included many that were highly monetised, and ‘gifts’ were given by patients not only to empirics but also to physicians.^68^ Significantly, the offer of healing services without payment accorded with Christian teaching, as the two patron saints of all healers, *Agioi Anargyroi*, refused monetary payment for their services, as their name suggests.^69^ More practically, most empirics perhaps refused monetary payment because the acceptance of cash could lead to incrimination in the event of prosecution by the state. To a degree, therefore, foregoing cash payment was a way for empirics to protect themselves from prosecution in order to be able to continue practising.^70^

As important to the empiric as any ‘payment’ was the acquisition of significant social standing/capital in their society – a standing that would have modified substantially the everyday life and economic opportunities available to an individual.^71^ As Mikes Paidouses and others have indicated, such a standing could be equal to, and at times higher than, that of a physician.^72^ While the emphasis of the existing studies on the absence of payment or the ‘symbolic’ payment given to empirics, which is seen also in discussions of African traditional medicine, is partly due to an idealised view of the rural past emanating from folklore studies,^73^ it also results from an implicit juxtaposition of the ‘gifts’ given to empirics with the requirements of physicians: monetary payment. Such payments were particularly difficult to provide in the Greek rural economy, even in the early 1950s: ‘In January I went to Makris [physician’s name], may he rest in peace, he was a good friend of ours; we did not have a penny left because we did not have insurance, all [our cash] was taken by Makris, we did not have insurance.’^74^ Paidouses, along with Richard and Eva Blum, emphasised the significant expense involved in seeing a physician, while interviewees indicated that some physicians financially exploited the villagers: ‘[The physician] was taking from you all you had.’^75^ In contrast, empirics did not demand monetary payment and, arguably, partly because of this, received instead elevated social standing within their local society and sometimes beyond – as well as the aforementioned ‘gifts’ – while also simultaneously protecting themselves against the risk of prosecution.

## 4 Legality, Availability and Practitioner Relationships

There is sufficient evidence to suggest that empirics enjoying a good reputation existed and were employed in equal measure with trained physicians in the early nineteenth century.^76^ In 1834, only two years after the creation of the modern Greek state, its Bavarian administration established the rules of medical practice under which the Medical Council [*Iatrosynedrio*] was the only authority allowed to grant an individual with a licence to practise medicine in Greece.^77^ Each candidate had to both ‘prove through valid certificates’ that he had studied and pass oral, written and practical examinations set by the Council.^78^ By December 1834, all those practising without a licence were ordered to cease doing so,^79^ although a provision seems to have been made for those ‘already practising [surgery, pharmacology and midwifery] empirically’ to attend a School of Surgery, Pharmacology and Midwifery, which would provide them with a licence if they were successful in their studies.^80^ Attendance at the school was limited, however, partly because it was based in Athens and partly because of the illiteracy of most empirics.^81^ Significantly, those empirics who attended the school could, at best, practise as surgeons [*heirourgoi*], but not as physicians.^82^ In 1837 there were 130 licensed empirics in Greece, who had presumably attended the first run of the school, and eighty-five physicians.^83^ In the town of Hermoupolis, there were eleven physicians (not all necessarily practising on the island continually), five empirics, two surgeons and nine phlebotomists practising in the 1830s and 1840s. The neighbouring community of Ano Syros had two empirics.^84^ The possession of a licence defined the legality of any medical practitioner until 1939, when a new law decreed that a licence to practise would be automatically given to the graduates of the Greek [Athens] Medical School.^85^ After the implementation of the law, some empirics petitioned for the right to be given a licence to practise *gratis*, but none was given such a licence.^86^ Evidence from specific cases, such as those of Gymnasios and Nikolaos Theodorakis, suggests that such licences were not easily obtained, if ever.^87^

Between 1907 and 1923 the Greek state doubled its territory and population; despite this, Greece did not suffer from a lack of physicians, with the ratio of physicians to inhabitants ranging around 10:10 000 in the first half of the twentieth century. Their concentration in Athens and in other urban centres, however, was astonishing. While, in 1940, the ratio was 29:10 000 for the county that included Athens, for other counties it was below 2.5:10 000.^88^ Within Crete, in 1948, the same ratio was 17:10 000 for cities but 3.6:10 000 for the non-urban population.^89^ The government made more than one attempt from 1923 to improve the provision of rural areas with physicians, but all such efforts proved unsuccessful for quite some time,^90^ and it was not until well after 1950 that the situation began to improve.

Those few physicians who did accept a post in rural Greece rarely remained there for long, as working conditions were extremely harsh. For example, Petros Apostolides, a physician armed with a leftist ideology, took up a post in inter-war rural Greece in which he assumed responsibility for twenty-four villages situated so far apart that, he felt, he could not properly serve them all. So, having made an enormous effort to carry out his work, he abandoned his position and left for the nearest town on sick leave.^91^ Rural inhabitants with access to a physician or a hospital ‘nearby’ would have to either bring the physician to their homes on a mule, often walking for hours, or take the patient to the ‘nearest’ hospital, ‘on the mule five to six hours’; still others would have to – or chose to – travel to Athens.^92^ Even in the 1960s, just as in the 1930s, a newly qualified physician serving the rural community would have been acutely aware of the limited service he could offer, which would have comprised only a ‘consoling visit, a visit of support; you could not do many things’ because there were no medicines available locally. He had no instruments and, significantly, his training had left him lacking in practical experience.^93^ Moreover, as rural inhabitants had no confidence in young physicians, it seems that their presence in the countryside was largely decorative.^94^ The most significant ‘help’ that such a physician could offer would be to refer the patient to the nearest hospital, where he/she would be treated for a fee;^95^ in the absence of such facilities, the physicians were just as helpless as they believed empirics to be, a fact readily acknowledged by some of their number:^96^

A. There were two or three empirics, at various points [of the island] …. They had the empiric attribute/ability to cure some [illnesses]…and they would advise and I confess that they did good to/[benefited] the place because there were no scientists [physicians] to face the situation. But I will tell you a secret: even if there were physicians they would not have known how to face such illnesses.

Q. Did the empirics offer something to the people?

A. The empirics always offered to the people, as long as they were not money-driven.^97^

The presence of traditional empirical healers during the first half of the twentieth century cannot be quantified since their practice was illegal and because the state took action against them not collectively but individually, and only extremely rarely, but the references to their existence and practice are so numerous and come from such a variety of sources that there can be no doubt about their strong presence, especially in the countryside.^98^ In Crete, in 1948, it seems that in rural areas there were more bone-setters than physicians (the ratio of bone-setters to the general population was 1:2000 and that of physicians was 1:2750). ‘Herb-doctors’ appear to have been much less common, but were clearly present (with ratios of 1:48 040 in rural areas and 1:43 730 in the cities).^99^ Sevaste Haviara-Karahaliou, employing exclusively oral accounts from Chios, cites no fewer than sixty named individuals who acted as empirics, primarily in the first half of the twentieth century but also earlier; they specialised in different ailments and resided throughout the island.^100^ In 1951, as the folklorist Polyd. Papahristodoulou emphasised in his speech on behalf of the Folklore Society, the presence and use of empirics in rural Greece was a given: ‘Popular medicine is a common theme in our lives. And we have [all] been cured by a *giatrissa* or a popular healer in the rural life [in which] we grew up.’ He added that ‘popular or empirical healing is used even today in the remote areas of Greek lands’.^101^ The existence and, in many cases, the names of empirics have been widely documented in all the sources cited here that refer to popular medicine in Greece, ranging geographically from Thrace to Rhodes and from the Cycladic island of Syros to the Ionian island of Cephalonia.^102^ Still, as knowledge of the identities and abilities of popular healers depended entirely on reputation and word of mouth, such references cannot reveal the full extent of their presence.^103^

In the twentieth century, especially in rural areas, inhabitants would have had no difficulty in distinguishing between physicians and empirics. The former was invariably an outsider to the area, an educated urbanite who had to present his licence to the local authorities before settling there.^104^ In contrast, the latter was a local person, perhaps born in the village or of long settlement there. The empiric was also largely uneducated, even if he/she could read, and was unlicensed. Moreover, the kinds of healing practised by popular healers and physicians were very different.^105^

## 5 Protecting (Healing) Tradition

Western physicians’ invective against the illegal practitioners with whom they competed for patients has been the subject of much scholarly discussion. It is possible to trace similar attacks on empirics in Greece, made not only by physicians but also by educated individuals, but these are not abundant.^106^ At the same time, many references to popular healers made explicit connections between some of the methods employed by them and methods practised in ancient Greek medicine.^107^ The purpose of folklore studies in Greece, which flourished from the late nineteenth century, was exactly that, namely, to trace the links between contemporary rural practices and those of Ancient Greece, so ‘proving’ the existence of cultural continuity.^108^ In 1909 Nikolaos Polites, the creator of Greek folklore studies, argued that popular medicine should be one of the field’s main themes.^109^ So, in 1925, the professional medical journal *Klinike* published in its first year a series of research articles on Greek popular medicine. Stilpon Kyriakides, the author and director of the Folklore Archive, used the archive’s collections to shed light on popular medicine, routinely making connections between the folklore material and Ancient Greek practices.^110^ He also emphasised, however, the cultural gap that existed between educated physicians and overwhelmingly uneducated patients, the two having a different understanding of the origin and nature of disease, and added that ‘it was time that the physicians turned their gaze towards the Greek people and studied the way the populace [*o laos*] understands diseases and the means that [the populace] uses to avert or cure them, long before they seek the advice of the physician’.^111^ Kyriakides argued that it would be beneficial for physicians to learn about ‘popular beliefs concerning diseases’ in order to narrow the ‘chasm that exists between the people and the physician’, so that the latter could become more ‘able to advise and instruct [the patients] in a language comprehensible by them and if necessary, [the physician should] utilise the popular beliefs for the benefit of his therapeutic purposes’.^112^ The articles written by Kyriakides and his position towards popular medicine were of enormous significance: it was the restrained but explicit support of Kyriakides and other folklorists and local historians for popular medicine that partly averted the possibility of an aggressive discourse against popular healing.^113^

That is not to say that action against individuals who practised healing without a licence was never taken: the example of Monk Gymnasios (above) demonstrates that it was. Another healer, practising in an urban area and accepting payments, was arrested and taken to court in the 1950s.^114^ The famous Vlachos, who practised as a bone-setter for much of the twentieth century, was repeatedly taken to court.^115^ In an earlier case, a popular healer who practised in rural Leukada was turned in by the physicians of the town of Leukada but escaped prosecution because no witnesses came forward to testify against him.^116^ It is notable that the community that he was serving ‘protected’ him by refusing to testify against him; indeed, evidence for such protection comes in many instances and in diverse locations. For example, in the mid-nineteenth century, Aggelos Katerelos was practising in a village while also working as a teacher in British-governed Cephalonia. When Cephalonia became Greek territory in 1864, his healing practice became illegal. At times of changing power structures Greek politicians were able to remove those individuals employed by the previous administration. However, Katerelos’ position as a teacher was spared because ‘the villagers want him because he practises as a doctor’.^117^ In the cases of two other empirics who were included in Cephalonia’s medical list of 1872, a note was made next to each name to the effect that the empiric ‘does not practise [medicine] as an occupation but if he is asked, due to necessity, he will provide an opinion’.^118^ In this instance the tactful articulation by the local authorities of how the empirics operated – being sought by, rather than seeking, patients – protected them from persecution.

In yet another case in the village of Pyrgi, on Chios, the practitioner K. Keras and his successor Magkaikas were both persecuted by the authorities,^119^ but the former enjoyed the protection of the local society in which he practised. In 1915 – that is, as soon as the Greek state established itself on Chios – the community issued a certificate assuring the police, invariably represented by a non-local, that Keras’ only occupations were those of pottery maker and tobacco cultivator, and ‘no other’.^120^ Keras was not a native of the village or of the island, and it is possible that this was why he was persecuted in the first place. In the late 1950s, in mainland Greece, an empiric accused by the local physician of practising without a licence was arrested and spent a few hours in jail, but was released by the local policeman, whose wife had received treatment from him.^121^ Like Keras, this empiric was not a native of his village, although he had been resident there for decades.^122^ In contrast, other empirics who operated in the same area were never turned in,^123^ although when an itinerant woman appeared in the same village selling healing herbs, the pharmacist called the police immediately.^124^ It is thus apparent that protection was offered to esteemed empirics particularly if they were native to the village. But, even then, empirics would be jailed when patients died.^125^

In some situations, and as more physicians moved to, or near to, rural areas, physicians and empirics seem to have co-existed amicably. For example, during the majority of his career as a practitioner in a northern Chios village, Giannes G. Heilas, the third generation of renowned empirics in his family, encountered no physicians.^126^ In the later years of his practice, however, when physicians were available in the nearby town of Volissos, Heilas would send serious cases to the physicians while he would treat minor injuries, as ‘the expense of visiting a physician was considerable’.^127^ The physicians never attempted to have Heilas prosecuted, and he practised until his death in 1965.^128^ Local communities, too, were instrumental in determining both practitioners’ and physicians’ success, or otherwise. Not only did they ‘protect’ local empirics if he/she were valued, but incoming physicians, if they were to become established in such a community, had to be, or be willing to become, familiar with local ways of thinking about healing, or at least be tolerant and respectful of the unwritten rules of the community.^129^ For example, the two physicians who were established in the town studied by Blum and Blum stated that they were ‘impressed’ by the therapies offered by the empirics and that they accepted the concept of the evil eye and employed locals to have it ‘removed’. The trained midwife, though not a local, also declared her trust in empirics.^130^ Yet another physician working in a Cretan village in the early 1950s assured his patient’s family that he, too, would invite the priest to perform a blessing when someone was ill in his family, just as the family planned to do after his visit.^131^ Although none of these physicians expressed outright support for the empirics, their compliant attitude was necessary to give them a foothold in the locality where they practised. However, some physicians were prepared to go further: one, in trying to eliminate the cultural gap between himself and his patients, pretended to be an empiric, although this occurred in an urban environment where his true identity could be more easily kept secret. His spectacular success in attracting patients and the fact that he accepted payment meant that he was soon reported to the police and arrested, only to reveal to the court that he was a licensed physician. He explained that he had carried out the deception because when he had practised as a physician he had been unable to attract any patients.^132^

The protection afforded empirics by their communities, then, was a significant factor in allowing them to continue practising throughout the nineteenth and much of the twentieth centuries in spite of their legal status.^133^ Moreover, the association of popular healing with Ancient Greek healing practices, as articulated by eminent folklorists and others, including Greek physicians, constrained the medical establishment from lashing out publicly against popular healing.

## 6 Choosing a Practitioner

Since both empirics and physicians operated in Greece, a degree of patient choice was available, dependent largely upon location. In the first half of the twentieth century, empirics and physicians occupied largely separate ‘catchment’ areas, with the former being established in rural areas and the latter based mostly in urban areas, although some also visited nearby villages or treated rural patients who had come to urban centres for that purpose.^134^ On Chios, for example, the rather inaccessible northern villages were served by local empirics, whereas the towns and the southern villages were relatively well served by physicians.^135^ Not all communities had an empiric, so, for many people, a consultation with any practitioner involved travelling and inconvenience;^136^ but even when both a physician and an empiric were available, the patient’s choice varied from case to case: ‘My mother, who broke her leg, did not want to go to the physician. She went to an empiric.’^137^ Even in urban centres where not only physicians but also hospitals were available, as in Hermoupolis, for some people the possibility of seeking a physician’s assistance did not seem, at times, even to be considered, as one interviewee revealed when discussing the death of many of his own siblings as young children:

A. That’s what I would hear, the *giatrines*, the local ones. Because they existed in the past, a *giatrina* was then considered [to be] better than a physician is [considered to be] today.

…

Q. Wouldn’t they take [the child] easily to a physician?

A. It wasn’t so much…to take you to the physician. You would become twenty years old and you would have never been to the physician.^138^

But when this interviewee’s own infant son became ill in 1949 it was the physician he called, signalling how, over time, they had come to be accepted.^139^ Nevertheless, for some diseases, empirics continued to be preferred to physicians: ‘People then went more to the various empirics and believed in the mysterious herbs for venereal diseases rather than [going to] the physicians.’^140^

As Philip P. Argenti and H.J. Rose explain, in Chios, during the 1940s, rural people maintained a ‘prejudice’ against physicians.^141^ The cultural ‘proximity’ of empirics appears to have been of considerable importance to patients in their choice of healer, especially for women, for whom modesty was an important consideration.^142^ The physician Petros Apostolides describes the reaction of a 40-year-old female patient when he recommended an injection: she suggested that he make a small cut in her dress at the injection site to avoid her having to reveal her body.^143^ Richard and Eva Blum stressed that ‘[t]he folk-healers share the culture of the peasants and shepherds…. They have the same assumptions about man’s relation to man, nature, and the supernatural that guide the lives of the uneducated people of the region.’^144^ It is unsurprising, therefore, that a physician who practised in rural Greece in the 1960s felt strongly the cultural distance that existed between him and the villagers:

…someone had a stroke…do you know what the first question was? Not ‘what is the matter with the patient?’, which is the logical question to ask…[but] ‘will he become as he was?’ The answer was of course ‘no’. And then came the tragic, for me, reply. ‘Then doctor, let’s not do anything’.^145^

In late twentieth-century Greece, biomedicine was generally perceived as ‘modern’ and effective and empirical practice as old-fashioned; yet it is important to note that, as late as the mid-1970s, many women in places such as rural Crete chose to consult an empiric first and then, only if necessary, a physician, with the question ‘it might still work, why not try it?’^146^ Since consulting an empiric did not involve a physical examination, it is not difficult to comprehend the reluctance of women to visit the ‘culturally distant’ urban physician except as a last resort.

The distance between patients and physicians was increased, too, by their different understanding and interpretation of disease. The villagers studied by Blum and Blum had an understanding of diseases and their causes that was very close to that of the empirics; it downplayed the significance of contagion while also involving disorders that ‘doctors do not know’, such as the ‘wandering navel’, the ‘waist’ and the ‘lifting of the kidneys’ – diseases that the folklorist Nikolaos Polites referred to as ‘non-existent’.^147^ These types of condition were described by Enrique Perdiguero, in his discussion of popular medicine in Spain, as ‘culture-bound syndromes’.^148^ Even in the case of biomedically recognised diseases, physicians and patients used different names to refer to them.^149^ More generally, then, language was another separating force between patients and physicians. Physicians tended to use *Katharevousa*, the formal language in which they were educated, a version of Greek that would have been incomprehensible to most rural patients.^150^ This point was masterfully, if unwittingly, articulated by a physician who referred to ‘the foreign language that physicians speak in front of the patient so that he and his circle cannot understand the scientific or professional communications …’.^151^ In contrast, empirics and their patients spoke not only the same form of Greek but also the same local dialect.^152^

## 7 Discussion and Conclusions

The Greek case reveals a situation in which the legality and primacy of licensed physicians was institutionalised right from the beginning of the modern Greek state in the 1830s. Unlike in England and elsewhere, where distinguishing between a physician and a quack was not easy, in Greece, the distinction between physicians and popular healers was very clear, certainly in the twentieth century.^153^ However, even in England, the distinction between physicians and what Davies calls cunning-folk was easy to make in the nineteenth century.^154^ The numerical dominance of physicians was well established in early twentieth-century Athens and the main urban centres, but their presence in rural Greece was limited. The converse appears to be true for popular healers, but this does not mean that they did not also exist in Athens and urban centres: rather, if they did, little evidence of their existence survives.^155^ Moreover, the close ‘regulation’ of the training of new popular healers meant that their numbers remained, at best, stable. As a result, they did not pose an ever-increasing threat to physicians. In rural areas the state was not quick to prosecute empirics, partly because few other medical practitioners were available there but also because local communities protected their popular healers. Community support for healers is not apparently unique to the Greek case, although its existence is not made explicit in the relevant literature either in Greece or elsewhere. For example, in England, both Davies’ cunning-folk and Moore’s wise woman received support from community members. Davies describes how, when Ellen Hayward was taken to court in 1906 for practising magic, community members wrote to the court, the press and the local magistrates supporting her presence and practice of herbalism in the community and arguing that a stop should be put to her persecution in the ‘interest of [the] community’.^156^ Similarly, Moore repeatedly refers to the ‘trust’ conferred on the wise woman Nell Racker and the community’s loyalty to her.^157^

Although, as in Greece, non-trained practitioners continued to exist in the West, the reasons offered for their survival differ drastically from the ones offered here for Greece. Firstly, the literature implicitly and explicitly accepts that profit was the reason for the existence not only of quackery but also of most of the practitioners of alternative medicines in the West. But, in distinguishing between the illegal practitioners, different pictures emerge. So, while the nineteenth- and twentieth-century quacks operated for profit and advertised their wares and skills, cunning-folk, wise women and popular healers did neither of these things explicitly. In France, we are told, popular healers were not ‘*entreprenant*’.^158^ The cunning-woman Ellen Hayward, when taken to court in 1906, argued that she ‘never demanded payment’, although her clients invariably paid her. Related is, in all probability, the fact that physicians in England or in France did not undertake a ‘concerted attack’ on cunning-folk but instead persecuted quacks.^159^ As in Greece, the low ‘payment requirements’ of the popular healers, their cultural proximity to their patients and the protection of the communities in which they practised ensured the continuation of their existence into the mid-twentieth century and beyond.

The Greek case offers an alternative to the discussions of unlicensed practitioners found in the Western literature. But, at the same time, it echoes the findings of Moore and Davies, who established not only the survival of localised patterns of non-orthodox medicine in the late nineteenth and early twentieth centuries but also the survival of cunning-folk and the role they played as healing providers in nineteenth- and early twentieth-century England. Although the Greek empirics seem only very rarely to have practised magic exclusively – as some cunning-folk did – it was nevertheless an integral part of their practice. This paper has demonstrated, for Greece, not only the empirics’ survival but also how and why such a survival was sustained. It is apparent, moreover, that Greece was not unique in the possession of such popular healers, but rather very much in line with England, Spain (where healers were known as *curanderos*) and perhaps also Italy.^160^ While a wide variation in terminology and practices, as well as such healers’ elusive presence, accounts for their relative neglect hitherto by historians of medicine, the findings of this paper have highlighted the urgent need for further work on popular healing in the twentieth century.

Monk Gymnasios, the healer this paper began with, seems, in fact, to have been an exception to the way in which Greek empirics operated. First, his status as an (in)famous public figure attracted thousands of ‘patients’, caused physicians to feel threatened by his presence and led the state to get involved.^161^ Second, he lacked a fixed base which would have provided him with the unspoken but valuable protection that the community bestowed on its own trusted empirics. Telling is his decision, concurrent with that to stop practising in 1932, to publish his remedies, going against the unwritten rule of secrecy that governed the practice of empirics in Greece.^162^

## Footnotes

1.
A.H. Hamoudopoulos, ‘Idou e aletheia dia ton Kalogeron. Ta pseude ton agyrton katarreoun. Oute thaumata eginan, oute iaseis. Einai kairos na epemvoun ai arhai’, *Macedonia*, 3 August 1930, 6495, 1.

2.
Giorgos Maniates, ‘Paron kai parelthon. Kompogiannitika’, *Eleutheria*, 22 May 1964, 6041, 3; Anonymous, ‘O Kalogeros Gymnasios eperase hthes eis ten polin mas’, *Macedonia*, 30 August 1930, 6521, 1, 3; G.I. Koukas (ed.), *Gymnasios Lauriotes. O Kalogeros kai oi syntages tou* (Athens: Leon, 1975), 11–12.

3.
Koukas, *op. cit.* (note 2), 7–12.

4.
L.P., ‘Mia nea synomilia me ton Agion Gymnasion’, *Macedonia*, 24 May 1932, 7093, 3; *ibid.*, 12.

5.
See, for example, Matthew Ramsey, ‘Medical pluralism in early modern France’, 57–80; David Gentilcore, ‘Medical pluralism and the medical market place in early modern Italy’, 45–55; Gunnar Stollberg, ‘Medical plurality and medical pluralism in Germany (nineteenth and early twentieth century)’, 141–51; all in Robert Jütte (ed.), *Medical Pluralism. Past-Present-Future* (Stuttgart: Franz Steiner Verlag, 2013); Waltraud Ernst, ‘Plural medicine, tradition and modernity. Historical and contemporary perspectives: views from below and from above’, in Waltraud Ernst (ed.), *Plural Medicine, Tradition and Modernity, 1800–2000* (London: Routledge, 2002), 1–18; Mark S.R. Jenner and Patrick Wallis, ‘The Medical Marketplace’, in Mark S.R. Jenner and Patrick Wallis (eds), *Medicine and the Market in England and Its Colonies, c. 1450–c. 1850* (Basingstoke: Palgrave Macmillan, 2007), 10.

6.
Robert Jütte, ‘Medical Pluralism in Early Modern Germany’, in Robert Jütte (ed.), *ibid.*, 27. As Loudon stressed, the term ‘qualified medical practitioner’ had no precise limits in England prior to the Apothecaries Act of 1815 and medical registration in 1858 (Irvine Loudon, “‘The vile race of quacks with which this country is infested”’, in W. F. Bynum and Roy Porter (eds), *Medical Fringe & Medical Orthodoxy 1750–1850* (London: Croom Helm, 1987), 106).

7.
Roger Cooter, ‘Introduction: The Alterations of Past and Present’, in Roger Cooter (ed.), *Studies in the History of Alternative Medicine* (Oxford: Macmillan Press, 1988), xiv–xv.

8.
Takahiro Ueyama, *Health in the Marketplace: Professionalism, Therapeutic Desires, and Medical Commodification in Late-Victorian London* (Palo Alto, CA: The Society for the Promotion of Science and Scholarship, 2010), 173–9.

9.
Eric W. Boyle, *Quack Medicine. A history of combating health fraud in twentieth-century America* (Santa Barbara, CA: ABC-CLIO, 2013), xvi.

10.
*Ibid.*, xxii; Michael Goldstein, ‘The persistence and resurgence of Medical Pluralism’, *Journal of Health Politics, Policy and Law*, 29, 4–5 (2004), 927–9.10.1215/03616878-29-4-5-92515602853

11.
S. King and A. Weaver, ‘Lives in Many Hands: The Medical Landscape in Lancashire, 1700–1820’, *Medical History*, 44, 2 (2000), 191; Boyle, *op. cit.* (note 9), xix, xxii; Roy Porter, ‘Before the Fringe: “Quackery” and the Eighteenth-Century Medical Market’, in Roger Cooter (ed.), *Studies in the History of Alternative Medicine* (Oxford: Macmillan Press, 1988), 6–7, 10–11.

12.
Matthew Ramsey, ‘Medical Power and Popular Medicine: Illegal Healers in Nineteenth-Century France’, *Journal of Social History*, 10, 4 (1977), 563.10.1353/jsh/10.4.56011632359

13.
Francesca Moore, “‘Go and see Nell; she’ll put you right”: The Wisewoman and Working-Class Health Care in Early Twentieth-century Lancashire’, *Social History of Medicine*, 26, 4 (2014), 695–714.

14.
*Ibid.*, 712.

15.
Owen Davies, ‘Cunning-Folk in the Medical Market-Place during the Nineteenth Century’, *Medical History*, 43, 1 (1999), 73.10.1017/s0025727300064711PMC104411010885133

16.
Violetta Hionidou, *Famine and Death in Occupied Greece, 1941–44* (Cambridge: Cambridge University Press, 2006), 29–31. For reasons of anonymity, all interviews conducted by the author are noted with a number. When names are used, these are pseudonyms.

17.
The professional collections were usually published in journals dedicated to folklore studies, such as *Laografika Symeikta*and *Laografia*, or to local studies, such as *Kefaleniaka Hronika*.

18.
Many of those are available online at Athens University, Folklore Collection, http://pergamos.lib.uoa.gr/; Aggelo-Dionyses Demponos, ‘Magike Iatrike ste Kefalonia. Therapeutika Xorkia’, *Kefaleniaka Hronika*, 1 (1976), 192–206.

19.
Exceptions exist, one such being the empiric studied by Clark (Patricia Ann Clark, *A Cretan Healer’s Handbook in the Byzantine Tradition: Text, Translation, and Commentary* (Farnham: Ashgate, 2011), 31–41). On the issue of the illegality of unlicensed healers, see the relevant section later in this paper.

20.
David Gentilcore, ‘Was There a “Popular Medicine” in Early Modern Europe?’, *Folklore*, 115, 2 (2004), 156–7, 160.

21.
*Ibid.*, 151–66.

22.
Ramsey, *op. cit.* (note 12), 581.

23.
Matthew Ramsey, *Professional and Popular Medicine in France, 1770–1830: The Social World of Medical Practice* (Cambridge: Cambridge University Press, 1988), 129–278; David Gentilcore, *Healers and Healing in Early Modern Italy* (Manchester: Manchester University Press, 1998), 1–26. But also see Mary Fissel, *Patient, Power and the Poor in Eighteenth-century Bristol* (Cambridge: Cambridge University Press, 1991), 63, who feels rather uneasy with some of the names used.

24.
Aglaia Mpimpe-Papaspyropoulou, *Paradosiake iatrike sten Peloponneso* (Athens: University of Athens, 1985), 51; Demetrios Oikonomides, ‘Demodes Iatrike en Thrake’, *Arheia Thrakikou Thesaurou*, 16 (1951), 188, 190; N. X. Razes *et al.*, *Anadrome. Gynaikeies morfes tou parelthontos me prosfora ston elleniko ygeionomiko horo* (Athens, 2008); Violetta Hionidou, “‘It was a bridge from life to death”: Hospitals during the Food Crisis, Greece 1941–44’, *Social History of Medicine*, 22, 2 (2009), 361–85; *Mykoniatika Hronika*, 4 February 1934, 3, referring to a female practitioner who was offering her ‘medical’ services in the 1880s in Ano Mera, Mykonos.

25.
Ramsey, *op. cit.* (note 12), 563–4. *Giatrines* is the plural form of *giatrina*.

26.
Mpampes Anninos, *Istorika semeiomata meta pollon eikonon* (Athens, 1925), written in 1897, 119–30.

27.
Clark, *op. cit.* (note 19), 16; Marlene Sue Arnold’s interviewee described himself as *empeirikos*, as did those of Blum and Blum (Marlene Sue Arnold, ‘Childbirth among Rural Greek Women in Crete: Use of Popular, Folk and Cosmopolitan Medical Systems’ (unpublished PhD thesis: University of Pennsylvania, 1985), 9, 131; Richard Blum and Eva Blum, *Health and Healing in Rural Greece* (Stanford, CA: Stanford University Press, 1965), 181, 189–94).

28.
At times, the term popular medicine is used to include domestic healing too.

29.
Another sizeable group was that of empirical midwives. Their activities will not be addressed here.

30.
Mikes Paidouses, ‘E laike iatrike eis ten Hion’, *Periodikon tou en Hio Syllogou Argente*, 6 (1964), 103–8; Richard and Eva Blum, *The Dangerous Hour. The Lore and Culture of Crisis and Mystery in Rural Greece* (London: Chatto & Windus, 1970), 370; Sevaste Haviara-Karahaliou, *E Laike iatrike tes Hiou* (Athens: Gasci-Hellas, 1993), 459 (oral account of E. Poules), 460.

31.
No. 3 Chios, male, born around 1909, rural residence; Blum and Blum, *op. cit.* (note 27), 198–205.

32.
No. 11 Mykonos, male, born in 1915, rural residence; No. 11 Syros, male, born around 1918, urban residence.

33.
Pantazes Kontomihes, *E Laike Iatrike ste Leukada* (Athens: Gregoris, 1988), 29; Theodoros I. Reginiotes, ‘E Matavase tes koinonias tou Kretikou horiou apo ten laike sten epistemonike iatrike’, in Anonymous (ed.), *Laike Iatrike, Diethnes Epistemoniko Synedrio Rethymno 8–10 Dekemvriou 2000, Praktika* (Rethymno: Istorike kai Laografike Etaireia Rethymnes, 2003), 514; Ioannes El. Volanakes, ‘Prolepse kai therapeia ton astheneon me votana sten Apodoulou Amariou Rethymnes Kretes’, in Anonymous (ed.), *Laike Iatrike, Diethnes Epistemoniko Synedrio Rethymno 8–10 Dekemvriou 2000, Praktika* (Rethymno: Istorike kai Laografike Etaireia Rethymnes, 2003), 69. See also K. Hrysanthe, ‘E semasia kai oi dikaiodosies tes Demodous Iatrikes’, *The Cyprus Medical Journal*, 3 (1950), 6, citing N. Polites, *Laografika Symeikta* (1920), 1: 5–6; Blum and Blum, *op. cit.* (note 27), 185–6. For an understanding of the range of ‘illnesses’ and conditions addressed by empirical healers, see Clark, *op. cit.* (note 19). As the effects of the ‘evil eye’ are considered to include a good number of physical afflictions, such as headaches and fatigue, I interpret here the efforts made to remove the ‘evil eye’ and its physical manifestations as healing.

34.
Hrysanthe, *op. cit.* (note 33), 15–19; Philip P. Argenti and H.J. Rose, *The Folk-lore of Chios* (Cambridge: Cambridge University Press, 1949), vol. 1, 385; Blum and Blum, *op. cit.* (note 30), 370, 144.

35.
See, for example, the photo of Vlachos while healing with a collection of icons prominently displayed behind him (Blum and Blum, *op. cit.* (note 27), jacket: 201).

36.
Virtually all sources referring to Greek popular medicine support this. See, for example, John Cuthbert Lawson, *Modern Greek Folklore and Ancient Greek Religion. A Study in Survivals* (Cambridge: Cambridge University Press, 1910), 21; Konstantinos I. Styliaras, *Sylloge laografikes yles (Laike Iatrike) ek tou horiou Dafnes Naupaktias, tou nomou Aitoloakarnanias*, 1970, http://pergamos.lib.uoa.gr/dl/object/uoadl:25883 (accessed 10 April 2015); Clark, *op. cit.* (note 19).

37.
On the classification of the untrained healers, see Ramsey, *op. cit.* (note 12), 562–3; Mpimpe-Papaspyropoulou, *op. cit.* (note 24), 51; Oikonomides, *op. cit.* (note 24), 188–90; Razes *et al.*, *op. cit.* (note 24); Hionidou, *op. cit.* (note 24), 361–85; *Mykoniatika Hronika*, 4 February 1934, 3, referring to a female practitioner who was offering her ‘medical’ services in the 1880s in Ano Mera, Mykonos; Euaggelia Kamme, ‘Mikra ethnografika meletemata tou topou mas’, *Mykoniates*, May 1992; Kontomihes, *op. cit.* (note 33), 53, 61–3; Argenti and Rose, *op. cit.* (note 34), 387.

38.
See, for example, Demponos, *op. cit.* (note 18), 196–206; Lawson, *op. cit.* (note 36), 21.

39.
For parallels see Elaine Leong and Sara Pennel, ‘Recipe collections and the currency of medical knowledge in the early modern “medical marketplace”’, in Jenner and Wallis, *op. cit.* (note 5), 137.

40.
Kontomihes, *op. cit.* (note 33), 51–63, 67–267, 308; Patricia A. Clark, ‘Landscape, Memories, and Medicine: Traditional Healing in Amari, Crete’, *Journal of Modern Greek Studies*, 20 (2002), 339, 344; Christos Papadopoulos, ‘Post-Byzantine Medical Manuscripts: New Insights into the Greek Medical Tradition, its Intellectual and Practical Interconnections, and our Understanding of Greek Culture’, *Journal of Modern Greek Studies*, 27 (2009), 107–30. See the recent publication of two such manuscripts translated into English (Clark, *op. cit.* (note 19); Barbara Zipser, *John the Physician’s Therapeutics. A Medical Handbook in Vernacular Greek* (Leiden: Brill, 2009)).

41.
Mikes Paidouses, ‘Demodes iatrike, empeirikoi iatroi’, *Periodiko tou en Hio Syllogou Argente*, 2 (1939), 60, referring to Chios. For an interesting discussion of the relationship between sorcery and Orthodox Church practice, see Charles Stewart, *Demons and the Devil: Moral Imagination in Modern Greek Culture* (Princeton, NJ: Princeton University Press, 1991), 222–43.

42.
Papadopoulos, *op. cit.* (note 40); Clark, *op. cit.* (note 40), 341–2.

43.
Clark, *op. cit.* (note 40), 353; Paidouses suggests that knowledge would pass on from father to the eldest son (*op. cit.* (note 41), 60), although Argenti and Rose mention that it was from mother to daughter (*op. cit.* (note 34), 428). In the Kostalas family, the son ‘inherited’ the bone-setting knowledge from his father while the daughter ‘inherited’ the midwifery practice from her grandfather’s sister (Stella Tsiropina (ed.), *O kyklos pou ekleise* (Chios: Omada Perivallontikes Ekpaideuses Gymnasiou Vrontadou 1999), 206). Blum and Blum, *op. cit.* (note 27), 183, 191, 193. A similar process of inheritance for *curanderos* is mentioned for Spain at the turn of the twentieth century and also for Italy (Enrique Perdiguero, ‘Magical healing in Spain (1875–1936): Medical pluralism and the search for hegemony’, 139; Sabina Magliocco, ‘Witchcraft, healing and vernacular magic in Italy’, 162–3, both in Willem de Blécourt and Owen Davies (eds), *Witchcraft Continued: Popular Magic in Modern Europe* (Manchester: Manchester University Press, 2004).

44.
Anninos, *op. cit.* (note 26), 123; Clark, *op. cit.* (note 40), 340, 353; Kontomihes, *op. cit.* (note 33), 50, 53; Mpimpe-Papaspyropoulou, *op. cit.* (note 24), 52; Raymond A. Mills, ‘Peasant Remedies from the Greek Islands’, *Bulletin of the History of Medicine*, 22 (1948), 441. Mills’ two practitioners had both been the fourth generation of practising empirics.18882858

45.
Arnold, *op. cit.* (note 27), 134.

46.
In the village studied by Blum and Blum, none of the local empiric’s children was interested in learning his art. Still, the empiric refused to teach his son-in-law because he was not a blood-relative and because he remarried soon after the death of the empiric’s daughter, and thus had behaved improperly (*op. cit.* (note 27), 194).

47.
Clark, *op. cit.* (note 19), 34; Alexandros Papadiamantis, *E Fonissa*, http://www.gutenberg.org/files/36205/36205-h/36205-h.htm, novella published in 1903.

48.
Mpimpe-Papaspyropoulou, *op. cit.* (note 24), 52–3; Blum and Blum, *op. cit.* (note 30), 162.

49.
See, for example, Mills, *op. cit.* (note 44), 441; Paidouses, *op. cit.* (note 30), 101; Oikonomides, *op. cit.* (note 24), 189; Clark, *op. cit.* (note 40), 353–4. Clark links secrecy to the fear of prosecution.

50.
Blum and Blum, *op. cit.* (note 27), 186, 192–3; Blum and Blum, *op. cit.* (note 30), 351; Stewart, *op. cit.* (note 41), 291. See similar situations described in Italy (Magliocco, *op. cit.* (note 43), 163); England (Owen Davies, ‘Charmers and Charming in England and Wales from the Eighteenth to the Twentieth Century’, *Folklore*, 109 (1998), 42–3); and the USA (Wayland D. Hand, ‘The Folk Healer: Calling and Endowment’, *Journal of the History of Medicine*, 26 (1971), 264).10.1093/jhmas/xxvi.3.2634938939

51.
No. 11d female, born in 1938 in a village, as adolescent moved to a town in Epirus, interview held in Athens; Oikonomides, *op. cit.* (note 24), 189; Lawson, *op. cit.* (note 36), 22, 49; see also the exasperated comment of a woman when she heard the charm being recited on the radio (C. Nadia Seremetakis, ‘Greek pains: subjectivity, material experience and communication in modernity’, in Charles Stewart (ed.), *Colonizing the Greek Mind? The Reception of Western Psychotherapeutics in Greece* (Athens: DEREE – The American College of Greece, 2009), 40).

52.
Paidouses, *op. cit.* (note 30), 78, 105; Blum and Blum, *op. cit.* (note 27), 189. Blum and Blum discuss one empiric, aged 76 years, who practised healing exclusively. This may have been due to his old age (*op. cit.* (note 27), 193). Ramsey describes a similar situation among early nineteenth-century French popular healers (*op. cit.* (note 12), 565).

53.
See, for example, the female empirics in Blum and Blum, *op. cit.* (note 27), 182–3; No. 3 Chios, *op. cit.* (note 31).

54.
However, even in the West charmers, ‘by tradition’, would not take cash payment (Davies, *op. cit.* (note 50), 44–5).

55.
No. 7d male, physician, born abroad, practised medicine on Chios from the early 1950s onwards. See also Clark, *op. cit.* (note 40), 354; Kontomihes, *op. cit.* (note 33), 29; Paidouses, *op. cit.* (note 30), 79.

56.
Mpimpe-Papaspyropoulou, *op. cit.* (note 24), 54, 56.

57.
Anninos, *op. cit.* (note 26), 129, quoting from *Ephemeris ton Athenon* 1825. Because the empiric’s patient died, he was ordered to stop practising.

58.
Zafeires Vaos, *Mageies – Getemata. Laike Therapeutike tes Melou (18os–19os ai.)* (Athens: F. Tsirones, 1982), 18, 17.

59.
G. Fasoulake, ‘E Hiake laike iatrike’, *Fone tes Hiou* (1931), 5.

60.
The payment was foodstuffs, cloth and some cash, all of which she used to pay for the boat ticket to reach home (Papadiamantis, *op. cit.* (note 47)).

61.
No. 3 Chios, *op. cit.* (note 31).

62.
M. Moustride, *Ta apomnemoneumata mou* (Athens: 1983), reproduced in Haviara-Karahaliou, *op. cit.* (note 30), 303.

63.
Elpinike Stamoule-Sarante, ‘Pos giatreuan ste Thrake. Giatrika, geteies kai hares’, *Thrakika*, 8 (1937), 351–3.

64.
Arnold, *op. cit.* (note 27), 135–6.

65.
No. 11d, *op. cit.* (note 51). Kontomihes also indicates that ‘they were paid for their service but rather symbolically’ (*op. cit.* (note 33), 29).

66.
Blum and Blum, *op. cit.* (note 27), 191, 163.

67.
A similar situation was in place in Italy (Magliocco, *op. cit.* (note 43), 163).

68.
Haralampos E. Krithares, *Anamneseis enos Giatrou* (Athens: Etaireia Kytheraikon Meleton, 1996), 147–8, referring to the late 1930s; No. 7d, *op. cit.* (note 55), mentioned by his wife, who was local. Physicians would receive ‘gifts’ in addition to their fee.

69.
Blum and Blum, *op. cit.* (note 27), 169; Mpimpe-Papaspyropoulou, *op. cit.* (note 24), 53; Stewart, *op. cit.* (note 41), 226.

70.
See also Ramsey’s comment indicating a similar situation in early nineteenth-century France (*op. cit.* (note 12), 566).

71.
Clark, *op. cit.* (note 40), 354; Kontomihes, *op. cit.* (note 33), 29; Clark, *op. cit.* (note 19), 17; Blum and Blum, *op. cit.* (note 27), 184; Mpimpe-Papaspyropoulou, *op. cit.* (note 24), 58. The empiric Vlachos was referred to as ‘Vlachos the God’ (Blum and Blum, *op. cit.* (note 27), 180). The Murderess, Papadiamantis’ protagonist, had almost open access to people’s houses only because she was a *giatrissa* When in need herself, she sought assistance from a woman she had helped in the past with her medical knowledge. The help that the Murderess received was articulated as a ‘return’ for the assistance she had given to the woman (Papadiamantis, *op. cit.* (note 47)). A similar observation is made by Ramsey, *op. cit.* (note 12), 570.

72.
Paidouses, *op. cit.* (note 30), 78. Rena Molho (ed.), *Oi anamneseis tou giatrou M. Yioel* (Athens: Patakis, 2010), 2nd edn, 33–5. M. Yioel refers to Thessaloniki around 1900 – that is, when it was still part of the Ottoman Empire. He states that the lay population was negative towards the qualified physicians, preferring the empirics. The empirics would usually charge more than the physicians (Molho, *ibid.*, 3–4).

73.
Walter Bruchhausen, ‘Medical pluralism as a historical phenomenon: a regional and multi-level approach to health care in German, British and independent East Africa’, in Anne Digby, Waltraud Ernst and Projit B. Mukharji (eds), *Crossing Colonial Historiographies* (Newcastle upon Tyne: Cambridge Scholars, 2010), 109.

74.
No. 22 Syros, wife and husband, wife born in 1927, rural Syros, referring to the late 1940s and early 1950s.

75.
No. 13 Chios, female, born in 1920, rural residence, referring to the 1940s; Paidouses, *op. cit.* (note 30), 74; Blum and Blum, *op. cit.* (note 27), 155–6; No. 24 Mykonos, female, born in 1915, resident of Mykonos-town; Elene Doundoulake and Nikos Hristinides, ‘Morfes laikes iatrikes periohes Arhanon Erakleiou Cretes’, in Anonymous, *op. cit.* (note 33), 220.

76.
Athanasios Mpasdras, ‘Oi giatroi tou strategou Makrygianne’ (unpublished PhD thesis: University of Thessaloniki, 2000), 111, 128–53; Emmanouel I. Emmanouel, *Iatrosofia kai Tsarlatanoi* (Athens: 1938), 10; Pan. Kretikou, ‘O Mandragoras’, *Laografia*, 19 (1961), 8, referring to ‘an excellent empiric doctor and a surgeon’ in 1838; Stelios Hiotakes, *Gia mia koinoniologia ton eleutheron epaggelmaton. Epistemonike epaggelmatopoiese ton Iatrikon Yperesion* (Athens: Odysseas, 1994), 160; F.C.H.L. Pouqueville, *Voyage en Morée, aI Constantinople, en Albanie et dans plusieurs autres parties de l’empire Ottoman pendant les années 1798, 1799, 1800 et 1801* (Paris: Chez Gabon, 1805).

77.
Mpasdras, *op. cit.* (note 76), 110; Anonymous, ‘Iatrosynedrion’, *Megale Ellenike Egkyklopaideia* (Athens: Pyrsos, 1926), vol. 12, 829; *Asklepios*, 1, 5 (1836), Appendix includes all Royal Decrees related to Medicine and Hygiene in the Greek territories (thereafter *Asklepios*, Appendix), ‘Peri ton ede meterhomenon eis ten Elladan ten iatriken, heirourgian, odontiken, kteniatreian, farmakopoiian kai maieutiken’, Decree of 14 (26) May 1834, 11–12.

78.
*Asklepios*, Appendix, ‘Peri systaseos Iatrosynedriou’, Decree of 13 (25) May 1834, 13–15.

79.
General State Archives of Athens, Mykonos archive, Luta eggrafa, folder 20, no. 155, 30 December 1834, letter to the municipal authorities of Mykonos.

80.
*Asklepios*, Appendix, ‘Peri misthodosias ton melon tou Iatrosynedriou’, Decree of 18 (30) January 1835, 23–5.

81.
Dionysios Touliatos, *Arrosties, giatroi kai farmaka* (Athens: 1956), 297–8, 18; *ibid.*, 23–5.

82.
*Asklepios*, Appendix, ‘Peri systaseos theoretikou kai praktikou katastematos tes heirourgias, farmakeutikes kai maieutikes’, Decree of 18 (30) May 1835, 79–80.

83.
Ar.P. Kouzes, *To epistemonikon ergon tes en Athenais Iatrikes Etaireias kata ten proten autes pentekontaetia (1835–1885)* (Athens: A.H. Pergamale, 1927), 4.

84.
Andreas  Fragkides, *Istoria tes nesou Syrou* (Athens: Syllogos ton en Athenais kai Peiraiei Syrianon, 1975), 107–8.

85.
Arnold, *op. cit.* (note 27), 133.

86.
*Ibid.*, 137.

87.
L.P., *op. cit.* (note 4), 3; Koukas, *op. cit.* (note 2), 12; Clark, *op. cit.* (note 19), 31–7; *ibid.*, 137. But see the case of Vlachos in note 161.

88.
Vasileios Valaoras, *E Ygieine tou Choriou* (Athens: Ypourgeion Anoikodomeseos, 1945), 26. In 1940, the USA figures were 6.3 and 16.8 for the two states with the lowest and the highest number of physicians per 10 000 population (Isidore Altman, ‘Changes in Physician-population Ratios among the States’, *Public Health Reports (1896–1970)*, 76, 12 (1961), 1053).PMC192983913860741

89.
Leland G. Allbaugh, *Crete: A Case of an Underdeveloped Area* (Princeton, NJ: Princeton University Press, 1953), 158.

90.
Hrestos D. Zelides, *Axiologese ton protovathmion yperesion ygeias tou agrotikou plethysmou* (Athens: Agrotike Trapeza Ellados, 1989), 122, 136–7, 197. In 1956, the Greek government introduced the limited-time compulsory service of newly qualified doctors in rural Greece in an attempt to force qualified practitioners to practise there (Arnold, *op. cit.* (note 27), 17, citing Spyros Marketos and George Merikas, ‘Primary Health Care in Greece’, *World Health Forum*, 1981, 2, 63). This was rarely enforced until 1967, when the dictatorship did enforce the law (No. 5d, male, born in 1940, studied and lived in Athens; No. 6d, male, born in 1941 in a rural area but moved very soon after to Athens; No. 7d, *op. cit.* (note 55)).

91.
Petros Apostolides, *Osa Thymamai, 1900–69* (Athens: Kedros, 1983), 135.

92.
Referring to 1920s Cephalonia (Gerasimos Regatos, *E Ygeia tou paidiou ste laike mas paradose* (Athens: Dodone, 1992), 288–9); No. 11d, *op. cit.* (note 51), whose ill younger sister was taken from the village to the doctor in the nearest town in the late 1930s but died; No. 13 Mykonos, female, born in 1906, rural residence; No. 2 Mykonos, female, born in 1917, rural residence, who travelled from Mykonos to Athens in 1945 for her 4-month-old son to be hospitalised; No. 16 Syros, female, born 1923, urban residence, whose brother was taken to Athens from Syros in the early 1940s despite the existence of a hospital there.

93.
No. 5d, *op. cit.* (note 90), referring to the late 1960s and his time spent as a community physician in Aitoloakarnania. On the lack of practical training, see also No. 6d, *op. cit.* (note 90).

94.
Blum and Blum, *op. cit.* (note 27), 147.

95.
Although this was considered a nominal fee by the physicians, it was seen as a major expense by the users. This was emphasised in many instances, as in the case of the interviewee whose baby daughter was hospitalised for a while in 1937: ‘I needed 100 drachmas per day for the hospital. 100 drachmas, that was a lot then!’ (No. 4 Chios, female, born around 1904, rural residence).

96.
See also Arnold, *op. cit.* (note 27), 217–19.

97.
No. 7d, *op. cit.* (note 55).

98.
Zelides, *op. cit.* (note 90), 113–15.

99.
Allbaugh, *op. cit.* (note 89), 158.

100.
Haviara-Karahaliou, *op. cit.* (note 30), 456–62. That this is not an exhaustive list is apparent from the fact that the empiric mentioned by my interviewee is not included (No. 3 Chios, *op. cit.* (note 31)).

101.
Polyd. Papahristodoulou, ‘E giatrike ste Thrake ton 18o aiona’, *Arheia Thrakikou Thesaurou*, 16 (1951), 166–80.

102.
*Ibid.*; Mills, *op. cit.* (note 44), 441–50, referring to the late 1940s and before; Hionidou, *op. cit.* (note 24), 361–85, referring to rural Syros in the early 1950s; Styliaras, *op. cit.* (note 36), 7.

103.
Clark, *op. cit.* (note 40), 354; Haviara-Karahaliou, *op. cit.* (note 30), 459 (oral account of E. Poules), referring to Abbot Anthimos Vagianos, whose ‘reputation had spread throughout the island and to neighbouring islands too’; Interviewee No. 11d, *op. cit.* (note 51), explained how the practitioner she visited asked her to ‘advertise’ his services ‘if she was cured’ after his treatment, something that the interviewee did because she was very happy with the outcome.

104.
*Asklepios*, Appendix, ‘Peri adeias tou epaggelesthai ten iatriken, heirourgian, farmakopoiian, kteniatriken, odontiken kai kteniatriken’, Decree of 7 (19) December 1834, 18–23.

105.
A read of the popular medicine recipes presented in the following publications demonstrates the variation within popular medicine and its distinctiveness from biomedicine (Clark, *op. cit.* (note 19); Argenti and Rose, *op. cit.* (note 34); Lampros Ap. Tatsiopoulos, *Laografika* (Ioannina: 1980), vol. 1, 52–72); Styliaras, *op. cit.* (note 36).

106.
No. 3d, female, born in 1940, Athens. The interviewee was educated in Athens and Germany, practised in Athens; Fasoulake, *op. cit.* (note 59), 5; T. Triantafyllou, ‘Ekthesis peri tes ygeionomikes katastaseos tes perifereias tou ygeionomikou kentrou Attikovoiotias kata to 1937’, *Arheia Ygieines*, 2 (1938), 188.

107.
Oikonomides, *op. cit.* (note 24), 181–2; Argenti and Rose, *op. cit.* (note 34), 382–4; Antonakopoulos traces no fewer than five nineteenth- and twentieth-century publications discussing and linking one particular popular therapy with the corresponding ancient Greek one (Georgios N. Antonakopoulos, ‘Empirical Bone-setting in Greece’, *Deltos*, 25 (2003), 31). See also the discussion in Clark, *op. cit.* (note 40), 341–2.

108.
Michael Herzfeld, *Ours once More: Folklore, Ideology, and the Making of Modern Greece* (New York: Pella, 1986), ch. 5.

109.
Polites, *Laografika*, 9–10, reprint of N. Polites, *Laografia*, 1 (1909), 10.

110.
Stilpon Kyriakides, ‘Demodes Iatrike’, *Klinike*, 1, 1 (1925), 35–7; ‘Demodes Iatrike. Kallikantzaroi’, *Klinike*, 1, 6 (1925), 199–201; ‘Demodes Iatrike. Vrykolakes B’, *Klinike*, 1, 10 (1926), 304–5. Many more articles followed in the same year by the same author addressing different topics of popular medicine.

111.
*Ibid.*, ‘Demodes Iatrike’, *Klinike*, 1, 1 (1925), 35.

112.
Kyriakides, *op. cit.* (note 111), ‘Demodes Iatrike’, *Klinike*, 1, 1 (1925), 36–7.

113.
A good number of similarly supportive articles were published in the 1930s and 1940s. See, for example, references in V. Skouvara, *Magika kai iatrosofika Eranismata ek Thessalikou Kodikos* (Athens: Akademia Athenon, 1967), 71–2.

114.
Blum and Blum, *op. cit.* (note 27), 233–4. This ‘empiric’ proved to be a licensed physician and thus was released.

115.
Blum and Blum, *op. cit.* (note 27), 199–200; http://fysiotherapeia.gr/heritage/ (accessed 19 April 2015).

116.
Kontomihes, *op. cit.* (note 33), 58.

117.
Gerasimos E. Pentogallos, *Giatroi kai Iatrike Kefalonias. Apo ta hronia tes Enosis mehri ton polemo tou 1940 (1864–1940)* (Thessalonike: University Studio Press, 2007), 369.

118.
*Ibid.*, 376.

119.
Paidouses, *op. cit.* (note 30), 104–5.

120.
Haviara-Karahaliou, *op. cit.* (note 30), 380–81. See also Kontomihes for a similar case in late nineteenth-century Leukada (*op. cit.* (note 33), 58).

121.
Blum and Blum, *op. cit.* (note 27), 189, 140.

122.
He was a refugee from Asia Minor who had on purpose amended his accent but nevertheless was called ‘Turk’ (*ibid.*, 189).

123.
Blum and Blum, *op. cit.* (note 27), 189, 140. On the protection the community affords to its own members against ‘outsiders’, in general rather than specifically in terms of healing and physicians, see the detailed case presented by Roger Just, *A Greek Island Cosmos* (Oxford: James Currey, 2000), 250–54.

124.
Blum and Blum, *op. cit.* (note 27), 149.

125.
See, for example, the case of Nikoles Kostalas, who was jailed for twenty-two days because one of his patients died (oral account of his daughter, Tsiropina, *op. cit.* (note 43), 206).

126.
Paidouses, *op. cit.* (note 30), 106; Haviara-Karahaliou, *op. cit.* (note 30), 464–5 (from a manuscript of Heilas’ son). See also Argenti and Rose, *op. cit.* (note 34), 421.

127.
See also Arnold, where her empirical interviewee makes a similar statement (*op. cit.* (note 27), 135, 219); No. 2 Syros, male, born in 1924, urban residence; No. 22 Syros, *op. cit.* (note 74).

128.
Haviara-Karahaliou, *op. cit.* (note 30), 464–5. Similar co-existence and co-operation in the 1950s is described by Regatos (Regatos, *op. cit.* (note 92), 287); Arnold, *op. cit.* (note 27), 135; Blum and Blum, *op. cit.* (note 27), 190.

129.
See Ramsey, making a similar point for early nineteenth-century France (*op. cit.* (note 23), 13).

130.
Blum and Blum, *op. cit.* (note 27), 140–43.

131.
Significantly, the physician is quoted speaking the local dialect, indicating his minimal cultural ‘distance’ from the patient’s family (Reginiotes, *op. cit.* (note 33), fn. 23, 517).

132.
Blum and Blum, *op. cit.* (note 27), 233–4.

133.
For example, No. 11d, *op. cit.* (note 51), referred to a bone-setter whose wife was trying to convince him that he should send the patients to physicians (referring to 1970s–1980s rural mainland Greece). Similarly, the family of Foteine Nikolaou-Giannakle feared prosecution and encouraged her to abandon the practice (referring to the post-Second World War era) (Razes *et al.*, *op. cit.* (note 24), 64–6).

134.
No. 3 Chios, *op. cit.* (note 31), indicated that a contracted physician was visiting a number of the southern villages offering his services there at regular intervals; No. 22 Syros, *op. cit.* (note 74).

135.
Paidouses, *op. cit.* (note 30), 74; Argenti and Rose, *op. cit.* (note 34), 421; Valaoras, *op. cit.* (note 88), 26; Sevaste Haviara-Karahaliou, *Giatroporemata ton ommation* (Chios: Medical Society of Chios, 2003), 99.

136.
No. 5d, *op. cit.* (note 90).

137.
Maria Maniate, aged 55, referring to her mother, aged 84 (Sofia P. Leptopoulou, *Laografika apo to Kranidi* (Kranidi: Municipality of Kranidi, 2001), http://argolikivivliothiki.gr/2009/04/04/λαϊκή-ιατρική-στο-κρανίδι-αργολίδας/ (accessed 23 June 2014), presumably referring to the late twentieth century. Also see No. 3d, *op. cit.* (note 106), referring to the late 1960s, when a patient asked to be discharged from the hospital in order to be treated by a local empiric; the same interviewee also referred to Vlachos (as also reported by Blum and Blum, *op. cit.* (note 27), 198–205).

138.
No. 11 Syros, *op. cit.* (note 37). A similar comment on not needing a physician, up to the age of forty in this case, was made by interviewee No. 12 Mykonos, female, born in 1913, rural residence.

139.
No. 11 Syros, *op. cit.* (note 37).

140.
Apostolides, *op. cit.* (note 91), 52.

141.
Argenti and Rose, *op. cit.* (note 34), 382; a similar comment by interviewee No. 2 Syros, male, born 1924, urban residence, referred to the early 1940s.

142.
See Enrique Perdiguero, ‘Healing alternatives in Alicante, Spain, in the late nineteenth and late twentieth centuries’, in Marijke Gijswut-Hofstra, Hilary Marland and Hans de Waardt (eds), *Illness and Healing Alternatives in Western Europe* (London: Routledge, 1997), 216–17.

143.
Apostolides, *op. cit.* (note 91), 129.

144.
Blum and Blum, *op. cit.* (note 27), 181; see also Ramsey, *op. cit.* (note 12), 578; similarly, in Gentilcore’s findings for early modern Italy, such a relationship was horizontal (*op. cit.* (note 20), 162).

145.
No. 5d, *op. cit.* (note 90). The same interviewee mentioned, again in amazement, that he attended at a labour which took place in a stable, a common occurrence in that village.

146.
Arnold, *op. cit.* (note 27), 200, 217–18.

147.
Blum and Blum, *op. cit.* (note 27), 132–5, 170–73; N. Polites, ‘Laografia’, *Laografika Symmeikta*, 1 (1920), 9–10. On the causes, see also Euaggelia K. Fragkaki, *E Demodes Iatrike tes Kretes* (Athens: 1978), 23–4.

148.
Perdiguero, *op. cit.* (note 142), 216–17. See Gentilcore, *op. cit.* (note 5), 51–2, referring to tarantism.

149.
Demetra Spitha-Pimple, ‘Morfes Laikou politismou: Laike iatrike’, in Anonymous (ed.), *op. cit.* (note 33), 546–52; Doundoulake and Hristinides, *op. cit.* (note 75), 174–7; Oikonomides, *op. cit.* (note 24), 183–8; Christos Lionis *et al.*, ‘Making doctor–patients communication more effective: disease names (terms) in the local dialect of a primary health care area in rural Crete, Greece’, *Patient Education and Counselling*, 34 (1998), S77.

150.
On the issue of language, see Peter Mackridge, ‘Katharevousa (c. 1800–1974). An obituary for an official language’, in Marion Sarafis and Martin Eve (eds), *Background to Contemporary Greece* (London: Merlin Press, 1990), vol. 1, 25–51. *Demotiki*, which is akin to vernacular, has been the official language of the state since 1976. On how little rural patients understood of what the physicians advised them, see Blum and Blum, *op. cit.* (note 27), 110–11, 204; Arnold, *op. cit.* (note 27), 223–32.

151.
Hrysanthe, *op. cit.* (note 33), 21.

152.
See Demetrios Karamperopoulos, ‘To Periodiko “Akademaike Iatrike” tou 1943 gia ten Demotike glossa sten Iatrike’, *Arheia Istorias Epistemon Ygeias*, 1 (2009), 61–7.

153.
Ueyama, *op. cit.* (note 8), 173–9; Boyle, *op. cit.* (note 9), xvi. Ramsey suggests that the types of healing practised by physicians and popular healers in early nineteenth-century France were recognised as ‘radically different’ by the physicians themselves. Nevertheless, he does not indicate whether the distinction was clear to all (*op. cit.* (note 12), 574).

154.
Davies, *op. cit.* (note 15), 55–6.

155.
Empirics never advertised their practice in the press, in contrast to physicians (see, for example, the newspapers *Aigaion*, Naxos, 18 March 1905, 4; *Scrip*, 1 January 1900; *Acropolis*, 28 September 1930).

156.
Owen Davies, *Cunning-Folk. Popular Magic in English History* (London: Continuum, 2003), 188, 191.

157.
Moore, *op. cit.* (note 13), 707, 708, 712. On trust, see Marijke Gijswut-Hofstra, Hilary Marland and Hans de Waardt, ‘Introduction. Demons, diagnosis and disenchantment’, in Gijswut-Hofstra, Marland and de Waardt, *op. cit.* (note 142), 8–9.

158.
Ramsey, *op. cit.* (note 12), 570.

159.
Davies, *op. cit.* (note 156), 165; Moore does not refer to any instances of prosecution of the wise woman she studied, despite the illegality of some of her actions (*op. cit.* (note 13)); Ramsey, *op. cit.* (note 12), 567, 574, 578.

160.
Perdiguero, *op. cit.* (note 142), 216–17; Perdiguero, *op. cit.* (note 43), 133–50; Magliocco, *op. cit.* (note 43), 151–73.

161.
Touliatos, *op. cit.* (note 81), 260. This was actually repeated in the 1950s with the renowned empiric Vlachos, who did obtain a licence but under the condition that he would operate under the supervision of a physician. Indeed, a physician was appointed, but he acted as Vlachos’ assistant (Blum and Blum, *op. cit.* (note 27), 198–205).

162.
Koukas, *op. cit.* (note 2), 12.

